# Testicular Adrenal Rest Tumors in Congenital Adrenal Hyperplasia: Study of a Cohort of Patients from a Single Italian Center

**DOI:** 10.3390/children10091457

**Published:** 2023-08-26

**Authors:** Rita Ortolano, Alessandra Cassio, Randa S. Alqaisi, Egidio Candela, Valeria Di Natale, Valentina Assirelli, Luca Bernardini, Elisa Bortolamedi, Erika Cantarelli, Beniamino Corcioni, Matteo Renzulli, Antonio Balsamo, Federico Baronio

**Affiliations:** 1 Pediatric Unit, IRCCS Azienda Ospedaliero-Universitaria di Bologna, 40138 Bologna, Italy; rita.ortolano@aosp.bo.it (R.O.); alessandra.cassio@unibo.it (A.C.); alqaisiranda@gmail.com (R.S.A.); valeria.dinatale@aosp.bo.it (V.D.N.); valentina.assirelli90@gmail.com (V.A.); federico.baronio@aosp.bo.it (F.B.); 2 Department of Medical and Surgical Sciences, Alma Mater Studiorum, University of Bologna, 40126 Bologna, Italy; antonio.balsamo@unibo.it; 3 Pediatric and Neonatology Department, Faculty of Medicine and Surgery, Mu’tah University, Alkarak 61710, Jordan; 4 Specialty School of Pediatrics, Alma Mater Studiorum, University of Bologna, 40126 Bologna, Italy; luca.bernardini10@studio.unibo.it (L.B.); elisa.bortolamedi@gmail.com (E.B.); erika.cantarelli@studio.unibo.it (E.C.); 5 Department of Radiology, IRCCS Azienda Ospedaliero-Universitaria di Bologna, 40138 Bologna, Italy; beniamino.corcioni@aosp.bo.it (B.C.); matteo.renzulli@unibo.it (M.R.)

**Keywords:** testicular adrenal rest tumors, congenital adrenal hyperplasia, CAH, pediatric endocrinology, 21-hydroxylase deficiency, pediatric oncology, differences of sex development

## Abstract

Testicular adrenal rest tumors (TARTs) are a common complication in male patients with congenital adrenal hyperplasia (CAH). The aim of our cross-sectional cohort study is to estimate the frequency of TARTs with the correlation of genotype and disease control on tumor development. Thirty-five male patients, aged 14–26 years, were included in the study, all followed by the same center of pediatric endocrinology in Bologna. We studied genotypes, hormonal profiles at different time intervals and testicular ultrasound. A logistic regression model with multivariant analysis was developed for the statistical analysis. TARTs were detected in 31.4% of the cases, 90.9% of them had a classic form with salt wasting, while 9.1% had a non-classic form. Additionally, a significant correlation between the incidence of TARTs and severity of genotype was detected. Patients with TARTs had markedly worse metabolic control on average (*p* = 0.027), reflected by high ACTH, 17OH progesterone, and overall delta4-androstenedione. In conclusion, a screening tool is mandatory, especially (but not exclusively) in patients with the most severe forms of CAH and poor endocrine control of the disease.

## 1. Introduction

Congenital adrenal hyperplasias (CAHs) are a group of autosomal recessive disorders of steroid synthesis that mainly involves adrenals. The most common form of CAH, responsible for about 95% of cases, is 21-hydroxylase deficiency (21-OHD) due to variations of *CYP21A2* [1]. According to the severity of 21-OH activity, three clinical forms are described: salt-wasting (<1% enzyme activity); simple virilizing (1–2%), which both are referred to as classic CAH; and non-classic (20–50%). The production of gluco- and mineralocorticoids depends on 21OH activity, therefore in the most severe forms, where the enzyme activity is less than 1%, the phenotype is characterized by adrenal insufficiency with salt wasting. In males with CAH, the loss of cortisol feedback on the hypothalamus and pituitary gland results in increased levels of the adrenocorticotropic hormone (ACTH). This hormone would continue to stimulate the adrenal cortex to become hyperplastic and to produce an excess of androgen hormones with genital virilization at birth in the 46, XX fetuses. In the non-classic forms, where adrenal cortical function is generally conserved, the hyperandrogenism is less severe and usually manifests in childhood as early pubarche with advanced bone age and early pubertal development in both sexes to acne, hirsutism and menstrual irregularity later in life in women. The incidence of severe forms is 1:15.000–1:30.000 live births, whereas the non-classic form is more common with an estimated incidence of 1:1000 births worldwide and even more frequent in some ethnic groups such as Hebrew Ashkenazi and Italians [2].

The clinical management mainly relies on glucocorticoid and mineralocorticoid treatment with the aim to substitute hormone deficiency in classic forms and to suppress ACTH production to reduce hyperandrogenism in all cases. The treatment is modulated on the severity of the enzyme deficiency and the level of endocrine control, with different goals during the life span [3].

Males affected by CAH are at risk of developing testicular adrenal rest tumors (TARTs), which is a not rare complication during follow up, with a prevalence that varies from 14 to 86%) [4].

The detection rate of TARTs has been reported between 5% and 35% by physical examination [5,6] and up to 86% by ultrasonography (US) screening, thanks to specific ultrasound features [7].

The etiology of TARTs is not completely understood. It has been hypothesized that TARTs originate from ectopic adrenocortical cells, which would migrate together with the descending testes, that during fetal life depends on ACTH and angiotensin II (AII) [8].

Another hypothesis is consistent with the possibility that the tumors originate from the proliferation of pluripotent fetal Leydig cells (FLCs) that contain ACTH, AII, and Luteinizin hormone (LH)/human Corionic Gonadotrophin (hCG) receptors: the presence of ACTH receptors on FLCs could explain the cell proliferation in males with CAH during fetal life due to chronic exposure to elevated ACTH levels. The simultaneous presence of LH/hCG receptors on tumors would also explain the significant increase in the TARTs’ prevalence during puberty, even in males with CAH despite good endocrine control of the disease [9].

The objective of this cross-sectional retrospective study is to estimate the frequency of TARTs, and evaluate the *CYP21A2* genotype and insufficient endocrine control as risk factors for the development of TARTs in a group of 35 males with CAH.

## 2. Materials and Methods

We retrospectively reviewed clinical charts and collected the clinical biochemical, genetic and radiological data of 35 pubertal males with a diagnosis of classic and non-classic 21-OHD regularly followed in the Pediatric Endocrinology Center of Bologna, age 14 and 26 years old (median age 19 years), who underwent testicular Ultrasonographic screening for TARTs between January 2015 and January 2020. Clinical and biochemical data were collected at the time of US and over the previous two years.

During follow-up, the patients were routinely evaluated in our center every 3–6 months. All cases were treated with hydrocortisone, adding fludrocortisone in those with salting wasting form. At each visit the equivalent dose of hydrocortisone taken by patients was calculated, expressed in mg/m^2^/day and adjusted according to clinical and endocrine control. Data regarding height, pubertal stage and endocrine control were retrospectively collected from files and clinical charts. During follow-up, height (cm) was measured with the Harpenden stadiometer which has a precision of 0.1 cm and weight (kg) was obtained by a steelyard scale, with an accuracy of 0.1 kg. From these measurements, the body surface (m^2^) was obtained according to the DuBois’s method. Stature and weight were then compared with the reference growth curves of Italian percentiles [10]. The evaluation of the pubertal stage in each patient was performed according to the Tanner’s stages. The difference between final height in males who had already reached it and the parent target was calculated (the latter corresponds to the sum of the average heights of the two parents +6.5 cm as male subjects). For males who had not reached their final height, we simply evaluated the difference between bone age and chronological age.

Delta4-Androstenedione (Δ4A) was measured by an immunochemiluminescence (CLIA) commercial kit Immulite 2000-XPi, 17-OHP was evaluated using radioimmunological assay kit DSL-5000, and testosterone levels were also measured by the immunochemiluminescence (CLIA) method via Access DXI 800 (Beckman Coulter^®^ Brea, CA, USA). Blood samples were taken after 8–10 h overnight fasting (at 8–9 am), before the morning dose of hydrocortisone.

Testicular ultrasound was performed in all males by means of Toshiba Aplio Canon^®^ (Ōtawara, Tochigi, Japan) machinery. In all cases, in addition, scrotal Eco-color-Doppler was performed, using high-frequency linear ultrasound probes (8–13 MHz) for soft tissues in order to search for any TARTs presence.

The preliminary B-MODE (gray scale) analysis was carried out with comparative morphological dimensional evaluation of the testicular ecostructure to investigate the presence of: microcalcifications (<3 mm), macrocalcifications (>3 mm), lesions focal points and their distribution within the testicles. A polylobulated margins formation, hypoechoic with respect to the remaining parenchyma with weak posterior acoustic reinforcement and increase in the vascular signal to the color-Doppler, is considered strongly indicative of TARTs. Due to the lack of sensitivity in the identification of microcirculation of the color-Doppler analysis, lesions ≤ 1 cm cannot be easily detected. In such cases, another methodological technique was performed by using a second-generation contrast agent (Sonovue^®^ Bracco sulfur hexafluoride at a dose of 4.8 mL) or multiparametric magnetic resonance (high-resolution weighted T2 morphological study, thin layer to 3 mm and weighted study in diffusion and perfusional study after infusion of paramagnetic contrast agent based on Gadolinium). 

### Statistical Analysis

Statistical analysis was conducted via the SPSS v23 platform for Windows (IBM Corp. Released 2013. IBM SPSS Statistics for Windows, Version 22.0. Armonk, NY, USA: IBM Corp). The first analysis carried out was used to verify the distribution of the variables. Most of the continuous variables were significant to the Shapiro Test showing a non-normal trend. The association between the discrete variables (Genotype and TARTs) was evaluated by the Chi square test and the Fisher exact test. Subsequently, in order to identify statistically significant differences between the continuous variables and the discrete variable (TARTs), the “U of Man-Whitney” and “*t*-tests for independent samples” tests were performed, respectively, for the variables distributed in no normal and normal ways. Univariate and multivariate regression analyses of factors related to the presence of TARTs were also performed. The parameters considered for the statistical analysis were genotype, metabolic control (ACTH, delta4 androstenedione 17-OH Progesterone, testosterone and their medians in controls at one and two years before and at the time of testicular ultrasound).

## 3. Results

### 3.1. Population

In all patients, except one, the diagnosis of CAH was made by newborn screening from the dried blood spot (DBS) performed at 48 h of life. Thirteen patients (37%) had the classic form with salt deficiency (SW), 12 patients (34%) had the classic form with simple virilizing (SV), and ten (29%) had the non-classic form (NC) of CAH.

In Table 1, the genotype and genotype group distribution of patients were reported.

Sonographic evidence of TARTs was detected in 11/35 patients (31.4%). Ninety-one percent (10/11) have the classic form of CAH with SW, while 9% of patients (1/11) have the NC form). In 100% of the cases, TARTs appeared at US as hypoechoic lesions, with a main axis between 4 mm and 20 mm (median value = 5 mm). Ten out of eleven patients (91%) presented lesions compatible with adrenal tumor at rest bilaterally. The median age at which the diagnosis of TARTs was made was 10 years.

### 3.2. Genotype Association in Patients with and without TARTs

The genotypes of the 11 patients with TARTs were distributed as follows: six patients (54.5%) had null mutations, four (36.4%) had group A mutations, and one patient (9.1%) had group C mutations.

**Figure 1:**
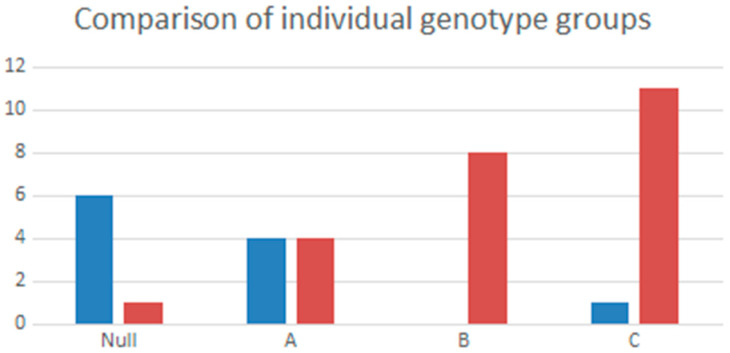
Comparison of single Null, A, B, C genotypes in patients with TARTs (in blue) and in the group without TARTs (in red).

In more detail, TARTs were significantly more present in patients with a Null genotype (asymptotic significance < 0.001) (Figure 1).

### 3.3. Endocrine Control

**Figure 2:**
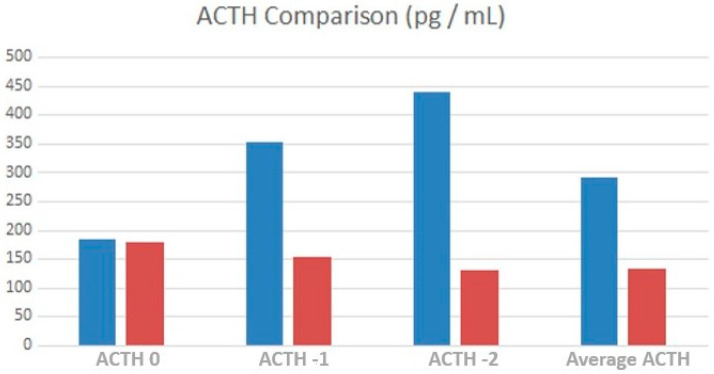
Comparison of average ACTH values, at time 0, one year and two years before testicular ultrasound between TARTs (blue) and non-TARTs (red).

ACTH level was significantly higher at T-2 in TART patients (*p* = 0.042), as shown in Figure 2.

**Figure 3:**
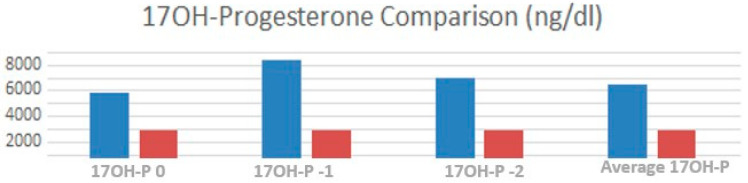
Comparison between the average 17OH-progesterone values, at time 0, one year and two years before testicular ultrasound between TARTs (blue) and non-TARTs (red).

The 17OH-progesterone levels were significantly elevated in TART patients at T-2 (*p* = 0.005). The average level of 17OH-progesterone was substantially higher (*p* = 0.036) in patients with TARTs compared to controls (Figure 3).

**Figure 4:**
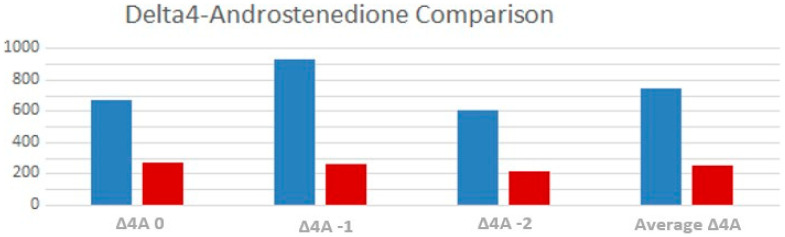
Comparison between the average Δ4A values, at time 0, one year and two years before testicular ultrasound between TARTs (blue) and non-TARTs (red).

Delta-4 androstenedione levels were significantly elevated in TART patients, both in T-2 (*p* 0.001) and T-1 (*p* = 0.006). Average value of delta-4 androstenedione was significantly higher (*p* = 0.02) in males with TARTs compared to the other patients (Figure 4).

The patients were subdivided into three groups according to their average endocrine control: group 1, with poor control (patients with levels of 17OH-P ≥ 2500 ng/dL, delta4-Androstenedione values higher than the normal range for sex and age); group 2, with good metabolic control (patients with 17OH-P levels between 200–2500 ng/dL, delta4-Androstenedione in the normal range); and group 3, patients with a suppressed hormonal profile due to pharmacologic overtreatment (17OH-P < 200 ng/dL, delta4-Androstenedione levels below the normal range). 

**Figure 5:**
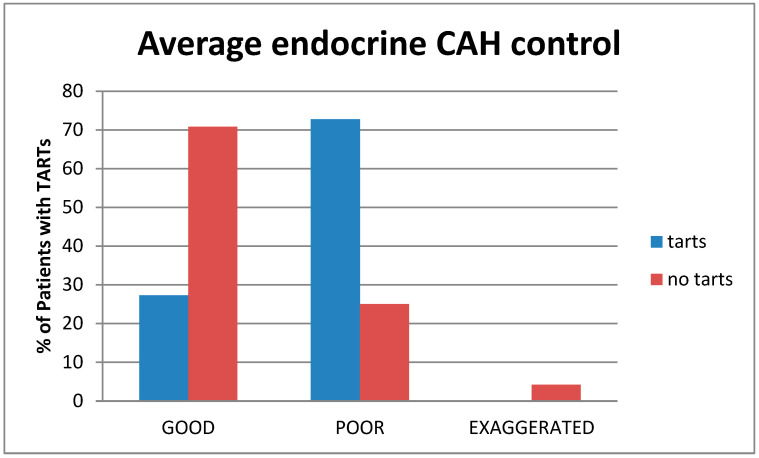
Comparison between the average endocrine control between TART (blue) and non-TART (red).

According to this division, 20 patients had average good control, 14 had poor control, while only one was averagely suppressed. Patients with TARTs had markedly worse metabolic control on average (*p* = 0.027) than the non-TARTs group (Figure 5).

Testosterone was not significantly higher in the group of patients with TARTs at any time of evaluation. 

### 3.4. Treatment

**Figure 6:**
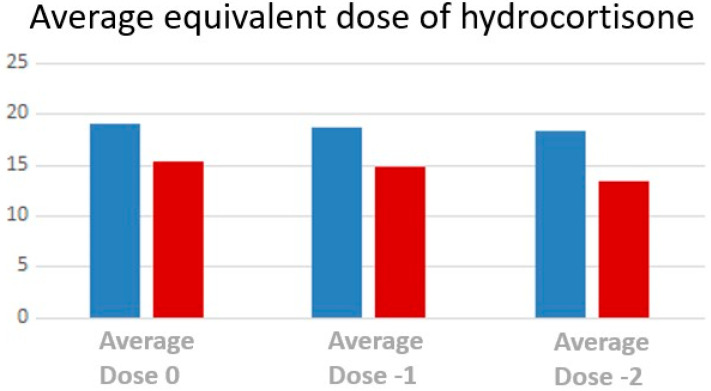
Comparison between the average equivalent doses of hydrocortisone for body surface area at T0, T-1 and T-2 for TART (blue) and non-TART (red).

Mean equivalent dose of hydrocortisone is significantly higher (Figure 6) in patients with TART at time T-2 (*p* = 0.004), at time −1 (*p* = 0.007), at time 0 (*p* = 0.028).

Specifically, the median equivalent dose of hydrocortisone for body surface was 18.92 mg/m^2^/die for patients with TARTs at time T-2 while 14.48 mg/m^2^/day for patients without TARTs. At time T-1, the mean F dose was 19.51 mg/m^2^/die in TARTs and 15.24 mg/m^2^/day without TARTs. Finally, at time 0, the median equivalent dose of hydrocortisone for body surface was 18.54 mg/m^2^/day for patients with TARTs while 16.02 mg/m^2^/die for patients without TARTs. 

We also carried out the same investigations, taking into consideration among our patients only those affected by the classical form (both SV and SW) divided in two groups, with and without TARTs.

The levels of ACTH and 17OH-P were not significantly different between the two groups.

**Figure 7:**
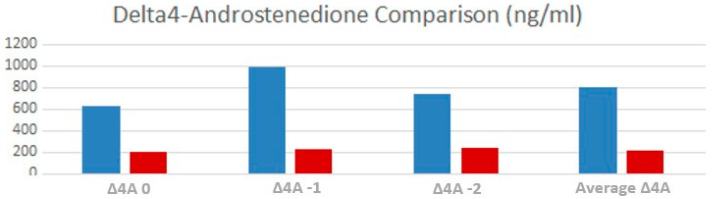
Comparison between the Delta4-Androstenedione values at T0, T-1 and T-2 for TART (blue) and non-TART (red).

Delta4-androstenedione levels (Figure 7) were considerably higher at times T-2 and T-1 (*p* = 0.001 and 0.005, respectively), but not at T0 (*p* = 0.08), whereas the delta4-androstenedione median level was significantly higher than those without (*p* = 0.005).

### 3.5. Logistic Regression and Multivariate Analysis

Logistic regression was performed, incorporating the following multivariant analysis: the coupled comparison between genotypes (Null and A vs. B and C), ACTH, 17OH-progesterone, and delta4-androstenedione. The values of delta4-androstendione at time-2 (*p* = 0.039) and genetic variations belonging to the genotypes Null and A (*p* = 0.002) remain significant in the multivariant analysis. In addition, this analysis has enabled us to calculate the odds ratio (OR) of the associated variables. At T-2, delta4-androstenedione is marginally associated with the onset of TARTs (OR = 1.004), whereas group B and C variations are inversely associated with the onset of TARTs (OR = 0.020).

## 4. Discussion

In our series of patients, the prevalence of TARTs was 31.4%. The data on the prevalence of TARTs in our region are interesting, especially since our region, Emilia-Romagna, one of the richest areas in Italy, has always been a pioneer in newborn screening and has been among the first regions in Italy to diagnose CAH at 48 h of life through DBS [11,12]. In Emilia-Romagna in particular, newborn screening for CAH was implemented as a pilot project from March 1980 to September 1983 and subsequently from February 1991 to today [13]. This prevalence data in our population, although subjected to newborn screening, therefore diagnosed at birth, being similar to the data of the populations not subjected to screening, allows us to be able to say that early diagnosis does not seem to be helpful in preventing the onset of TARTs. 

In a recent Turkish study, TARTs were detected only in 17.4% of the cohort of males with CAH and the youngest patient with a TART was 2 years old [14]. Based on what has been reported in numerous studies, it is known that the prevalence of TARTs is variable and higher among adolescents and young adults than among children. However, it has been also demonstrated that TARTs can occur in prepubescent individuals, particularly those with poor metabolic control [15,16,17,18]. Consequently, the prevalence of TARTs detected in our sample is likely to be highly dependent on the characteristics of our subjects that were older than 14 years and in the G3-4 pubertal stage. On the other hand, we cannot confirm if TARTs would be present also in prepuberty due to our cohort features.

As for the localization of the lesions, our findings are concordant with scientific literature regarding the bilateral distribution of TARTs that was confirmed in 90% of our patients. The data do not significantly deviate from the average proportion (77%) reported by Engels M. et al. [17].

The severity of the genetic variation correlates strongly with the presence of TARTs. In our cases, the variations on *CYP21A2* belonging to the null and A groups are most frequently associated with the onset of TARTs, as reported by literature [1]. The persistence of significance for the multivariate analysis of the strongly negative association (OR = 0.020) between genotypes B and C and the presence of TARTs further validates the importance of the genotype as a risk factor for TARTs. Nevertheless, several studies do not confirm this cause-effect hypothesis [4,15,17], possibly due to the paucity of the patients enrolled.

As far as we are aware, only 10 males with non-classic forms and TARTs have been described in the scientific literature to date [7,13,18,19,20,21,22]. The prevalence of TARTs in the NC-CAH could be undervalued due to the lack of data on large populations [23]. Our study confirms the possibility that although rare, it is also possible to find TARTs in cases with NC-CAH. It seems to confirm that TARTs may arise in cases without severe CAH, most probably growing more rapidly during puberty when LH increases and would act as a growth factor for TARTs that show LH/hCG receptors.

Moreover, we underline that in our cohort TARTs were not detected in subjects with SV form, differing from other studies where TARTs were identified in this group [6]. This most probably could be related to the small number of patients in our study.

Our results showed that males with TARTs had significantly worst average metabolic control, specifically two years before the diagnosis of TARTs. In particular, the multivariate analysis showed that only Δ4A at time −2 (OR = 1.004) is the persistently significant parameter associated with the presence of TARTs. Therefore, we have considered Δ4A the most dependable parameter due to its stability and its capacity to mirror the pathology’s metabolic control more closely. From this observation, we can infer that the presence of TARTs is correlated with metabolic control in our patients at least 2 years before the diagnosis, confirming the hypothesis that TARTs are more likely found in patients with severe CAH not in adequate and stable endocrine control [9,16].

Our results are in line with Mendes-Dos -Santos et al.’s study [15] that supports the importance of elevated Δ4A as a risk factor for TARTs. We observed that patients with TARTs received equivalent doses of hydrocortisone at noticeably higher concentrations than those without TARTs: it likely means that in these cases CAH were more severe and metabolic control is hard to achieve, so a higher dose of hydrocortisone was needed, once treatment non-adherence had been excluded. Conversely, our work also showed that not all TART-treated patients had insufficient metabolic control, just as not all TART-free patients always had great metabolic control.

Analyses of the studies by Mendes-Dos-Santos and Dumic et al. yielded comparable results, confirming that even though metabolic control may be a significant risk factor, the etiopathogenesis of these tumors is undoubtedly more complex and, in many respects, still unknown [15,16]. Also, in another study including males with *CYP21A2* deficiency, the relationship between poor metabolic control and the development of TARTs was not found [24].

Our data do not allow us to establish the ability of our patients to be fertile because, probably due to their young age, they have not had children yet. The lack of these data together with the lack of some specific analyses, for example spermiogram, FSH and LH values, does not allow us to make correlations between the size of the TARTs and their impact on the fertility. It is now well known that in males with CAH the fertility rate is reduced compared with the normal population, the most frequent cause being the presence of TARTs [25,26]. 

In case TARTs’ dimensions do not decrease with glucocorticoid therapy or in case of persistent azoo-spermia, surgical intervention may be considered [27]. Surgical intervention may be considered, but the effect on fertility is not yet known [28]. Moreover, in poorly controlled males, elevated ACTH levels induce high levels of androstenedione, partly aromatized to estrone. The high levels of these steroids suppress the hypothalamic–pituitary–gonadal axis, leading to hypogonadotropic hypogonadism [29].

The strengths of this study are multiple. First of all, patients recruited in the study have been followed by a single pediatric endocrinology center. Most of the diagnoses were performed by newborn screening, a key factor that allows us to have a population with homogeneous follow-up from birth. The clinical data are therefore homogeneous, and the hormonal tests were performed by a single laboratory. The patients were all subjected to testicular ultrasound by the same operator, with a consolidated awareness of TARTs, in order to eliminate the possibility of bias associated with operator-dependence of the ultrasound examination. All patients were also treated according to the same pharmacological approach, performed by the same medical team.

On the other hand, the limitations of this study include the limited number of patients and the inability to compare other parameters (such as FSH, LH, inhibin B, and spermiogram levels) between patients, as these hormones are not routinely administered during the follow-up. Furthermore, we do not have the follow-up data to support our hypothesis, i.e., that the poor metabolic control is the cause of the TARTs, and as the patients’ metabolic control improved, the TARTs disappeared or decreased in size.

## 5. Conclusions

In conclusion, our data confirm that the *CYP21A2* genotype could be considered in our cohort the primary risk factor for TARTs. Subjects suffering from the classic form with SW are the most frequently affected cases, although we also found TARTs in a male with a non-classic form. Inadequate metabolic control could be considered another significant risk factor, although we found TARTs in males with less severe forms of CAH and good control. Delta4-Androstenedione was the most reliable parameter due to mirroring the pathology’s metabolic control. Newborn screening does not appear to prevent the formation of TARTs. Since there is still no clear explanation of the etiopathogenetic mechanisms underlying the development of TARTs, we can confirm that a regular ultrasonographic screening would help to find TARTs particularly, but not exclusively, in those affected by severe forms of CAH and poor endocrine control.

## Figures and Tables

**Table 1:** Patients with corresponding phenotype and genotype. Null, A, B, and C are classified according to Wedell A’s classification. SV = Simple Virilization. SW = Salt Wasting.

Patient	Phenotype	Genotype	Genotype Group
1	CL (SW)	delB/delB	Null
2	CL (SW)	delB/delB	Null
3	CL (SW)	conv/delB	Null
4	CL (SW)	conv/delB	Null
5	CL (SW)	delB/delB	Null
6	CL (SW)	delB/delB	Null
7	CL (SW)	delB/delB	Null
8	CL (SV)	I172N/IN2 + Q318X	B
9	CL (SW)	IN2/delB	A
10	CL (SW)	IN2/Q318X	A
11	CL (SW)	IN2/R356W	A
12	CL (SW)	IN2/IN2	A
13	CL (SW)	IN2/delB	A
14	CL (SW)	IN2/R356W	A
15	CL (SV)	IN2/R356Q	A
16	CL (SV)	IN2/I172N	B
17	CL (SV)	I172N/delB	B
18	CL (SV)	IN2/I172N	B
19	CL (SV)	I172N/D8bp	B
20	CL (SV)	I172N/R356W	B
21	CL (SV)	IN2/I172N	B
22	CL (SV)	IN2/I172N	B
23	CL (SV)	I172N/ClE6	B
24	CL (SV)	conv(prom + P30L)/Q318X	B
25	NC	V281L/V281L + R366H	C
26	NC	IN2/NT2721	C
27	NC	V281L/V281L	C
28	NC	V281L/V281L	C
29	NC	V281L/V281L	C
30	NC	V281L/P30L	C
31	NC	NT2721/V281L	C
32	NC	V281L/IL72N	C
33	NC	IN2/V281L	C
34	CL (SV)	L446P/R356W	C
35	NC	IN2 + Q318X/V281L	C

## Data Availability

All clinical data and material are available in our Pediatric Unit.
